# Exploring the Conformational Landscape of Poly(l-lysine) Dendrimers Using Ion Mobility Mass Spectrometry

**DOI:** 10.1021/acs.analchem.4c00099

**Published:** 2024-05-30

**Authors:** Florian Benoit, Xudong Wang, Junxiao Dai, Niklas Geue, Richard M. England, Anthony W. T. Bristow, Perdita E. Barran

**Affiliations:** † Michael Barber Centre for Collaborative Mass Spectrometry, Manchester Institute of Biotechnology, Department of Chemistry, The University of Manchester, 131 Princess Street, Manchester M1 7DN, U.K.; ‡ Advanced Drug Delivery, Pharmaceutical Sciences, R&D, AstraZeneca, Macclesfield SK10 2NA, U.K.; § Chemical Development, Pharmaceutical Technology and Development, Operations, AstraZeneca, Macclesfield SK10 2NA, U.K.

## Abstract

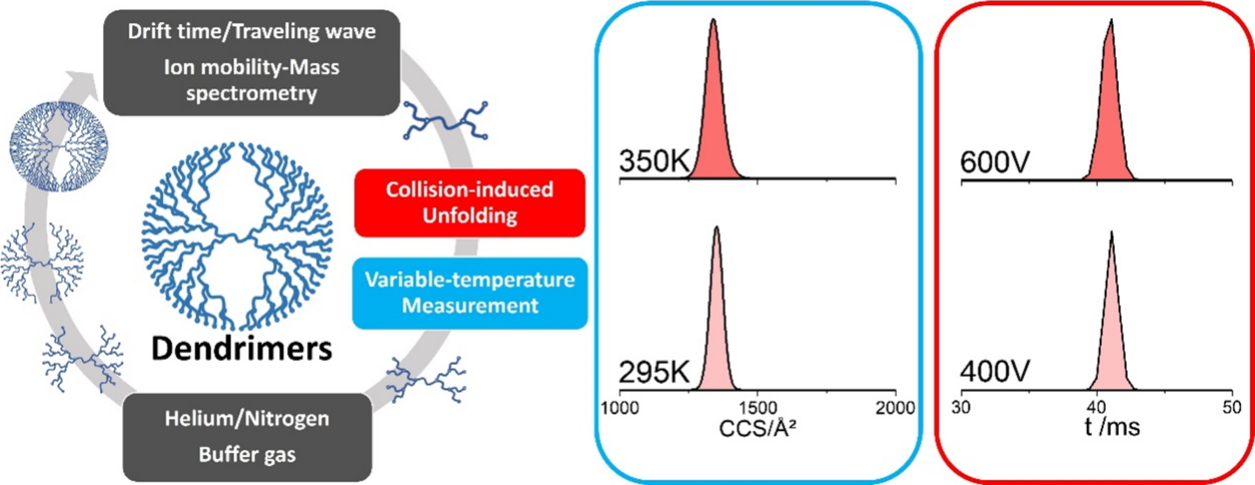

Ion mobility mass spectrometry (IM-MS) measures the mass, size, and shape of ions in the same experiment, and structural information is provided via collision cross-section (CCS) values. The majority of commercially available IM-MS instrumentation relies on the use of CCS calibrants, and here, we present data from a family of poly(l-lysine) dendrimers and explore their suitability for this purpose. In order to test these compounds, we employed three different IM-MS platforms (Agilent 6560 IM-QToF, Waters Synapt G2, and a home-built variable temperature drift tube IM-MS) and used them to investigate six different generations of dendrimers in two buffer gases (helium and nitrogen). Each molecule gives a highly discrete CCS distribution suggestive of single conformers for each *m*/*z* value. The ^DT^CCS_N_2__ values of this series of molecules (molecular weight: 330–16,214 Da) range from 182 to 2941 Å^2^, which spans the CCS range that would be found by many synthetic molecules including supramolecular compounds and many biopolymers. The CCS values for each charge state were highly reproducible in day-to-day analysis on each instrument, although we found small variations in the absolute CCS values between instruments. The rigidity of each dendrimer was probed using collisionally activated and high-temperature IM-MS experiments, where no evidence for a significant CCS change ensued. Taken together, this data indicates that these polymers are candidates for CCS calibration and could also help to reconcile differences found in CCS measurements on different instrument geometries.

## Introduction

The coupling of ion mobility to mass spectrometry (IM-MS) permits measurement of the size and shape of *m*/*z* selected molecules in a single experiment, which has widespread applications including characterization of newly synthesized compounds,^1,2^ discerning isobaric metabolites from complex mixtures,^3^ determining the structure and topology of biological complexes,^4^ and examining protein fold stability.^5,6^ IM-MS experiments measure the mobility of an ion in a given gas, and this value is commonly converted to and reported as temperature-dependent, rotationally averaged collision cross sections (CCS), which can be compared to literature data or to values computationally predicted from candidate geometries. Different forms of ion mobility instrumentation have been developed including traveling wave ion mobility spectrometry (TWIMS),^7,8^ more recently trapped ion mobility spectrometry (TIMS),^9,10^ and linear field drift tube drift tube ion mobility spectrometer (DTIMS).^11^ The first two require the use of calibrants in order to obtain CCS values from the experimental data, and even DTIMS instruments benefit from well-characterized standards in order to compare data across laboratories.^12^ As with all reference materials, the best calibrants bracket the range of observables of the target analyte ions, and for IM-MS, they will have at least a similar mass and range of charge states,^13^ and ideally will maintain structural rigidity upon exposure to different stimuli such as ionization source conditions, collisions with gas, and drift gas temperature.

Due to the increased use of IM-MS over the past 15 years, a range of calibrants have been proposed to enable the calculation of CCS values.^14,15^ For small, singly charged molecules, Agilent Tunemix (molecular weight ≈100–2800 Da), a series of polymers capped with an amino group or commercially available polyalanine, is widely used.^16^ For larger and more highly charged analytes, the most common type of molecule employed is readily available proteins; however, low charge states, representative of native folds, can present with more than one conformation and are prone to restructuring due to activation on transmission into the gas phase, injection into the drift cell, and also due to changes in the temperature of the drift gas.^14,17^ A more ideal calibrant suitable for higher charge state species would not deform under normal operation conditions and could be used as a system suitability test between different instruments.

**Figure 1:**
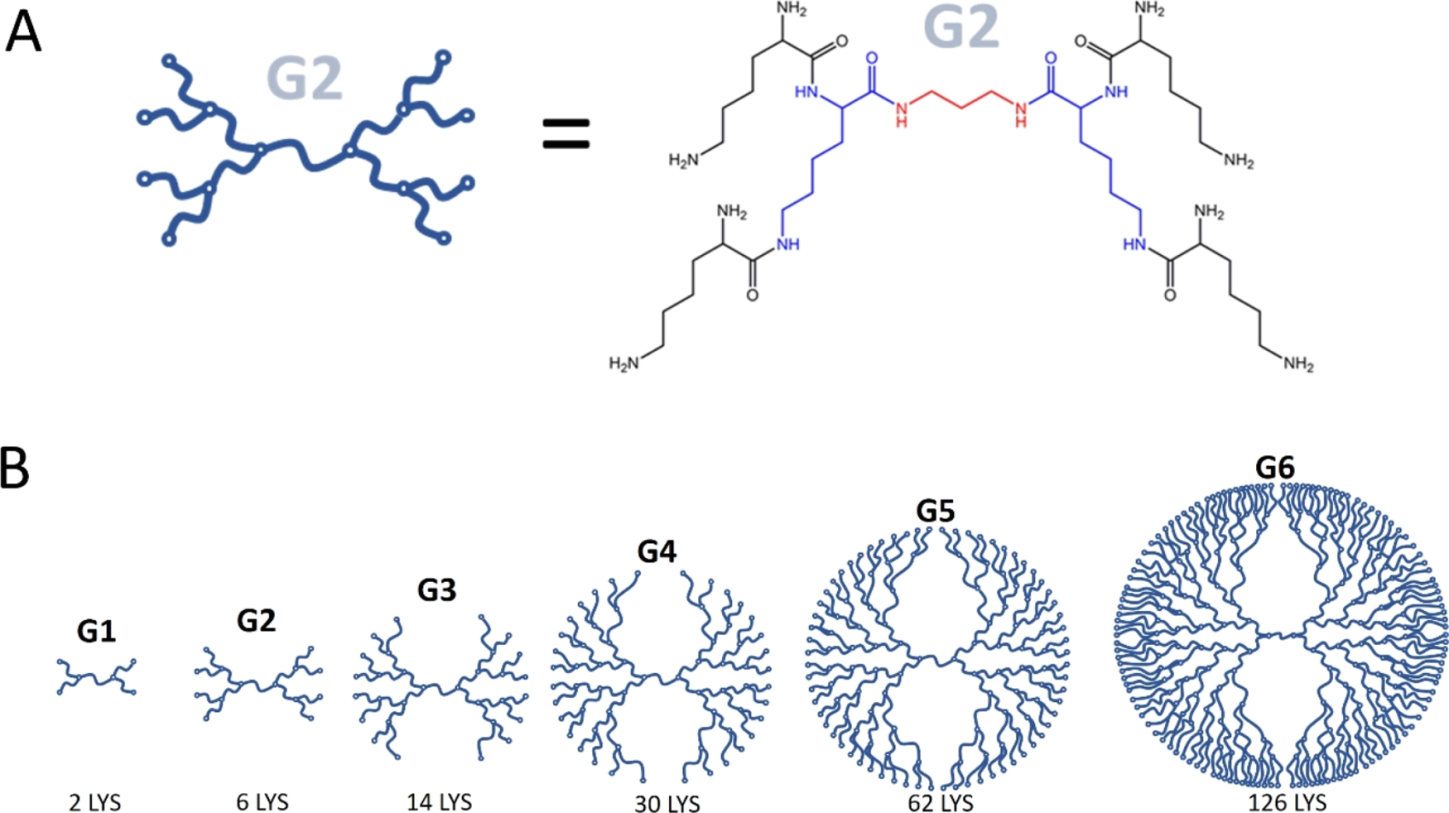
(A) Schematic structure and chemical structure of the G2 PLL dendrimer with the core (red) and the constituents of G1 (blue) and G2 (black). (B) Schematic of the G1-G6 PLL dendrimers including the total number of lysine units.

Dendrimers are polymeric molecules that are associated with several chemical application areas including in manufacturing,^18^ dyes,^19^ display technology,^20^ materials science,^21^ and particularly drug delivery.^22^ They are developed with controlled branching and permit tuning of the end groups via covalent or electrostatic interaction. This makes it possible to tune tuning their solubility, and controlling the molecular weight through dendrimer generations gives rise to monodisperse macromolecules.^23^ Polylysine dendrimers were first synthesized in the 1980s using methods by Denkewalter et al. and can be built up to the 10th generation.^24^ Later, Tam et al. reported conventional solid-phase peptide synthesis (SPPS) for divergent construction of a third-generation unsymmetrical polylysine dendron.^25^ Polylysine dendrons have been made on PEGA^26,27^ and TentaGel resins,^28^ where following purification and isolation, subsequent generations can be built using PEG as the hydrophilic tail.^29^ Supramolecular structures based on polylysine dendrons were first reported by Hirst et al.,^30^ who highlighted the effects of hydrogen bonding to form gel-like structures as well as characterizing properties, such as the ratio between the chirality of the dendrons^31^ and the length of the diamine spacer. As a result of the wide application scope for dendrimers, several analytical methods have been used to characterize them including NMR^32^ and size exclusion chromatography.^33,34^ In part due to their monodispersity and broad mass range (700–30,000 Da), dendrimers have previously also been proposed as MS calibrants, for example, in MALDI-MS^35^ and as IM calibrants for CCS calculations.^36^ Recently, Saintmot et al. used IM-MS, coupled to molecular dynamics simulations, to investigate the conformations of dendrimers^37^ and dendriplexes.^38^ Here, we investigated a family of polylysine dendrimers (Figure 1 and Table 1), where the number of surface amino groups (−NH_2_) doubled in number with the increasing generation, allowing for greater charge accommodation (e.g., generation 2 (G2) has eight surface groups, Figure 1A). We employed three different IM-MS instruments with two drift gases and reported on the measured charge states and CCS ranges under standard and activating conditions to determine if these molecular architectures could have applicability as ion mobility calibrants.

**Table 1:** Structural Parameters of Generation 1 to 6 PLL Dendrimers Including Molecular Formula and Monoisotopic Mass (*m*_mono_), as well as the Number of Lysine Residues (*n*_Lys_) and Surface NH_2_ Groups (*n*_NH_2__)

generation	molecular formula	*n*_Lys_	*n*_NH_2__	*m*_mono_ (Da)
G1	C_15_H_34_N_6_O_2_	2	4	330.3
G2	C_39_H_82_N_14_O_6_	6	8	842.7
G3	C_87_H_178_N_30_O_14_	14	16	1868.4
G4	C_183_H_370_N_62_O_30_	30	32	3918.9
G5	C_375_H_754_N_126_O_62_	62	64	8020.0
G6	C_759_H_1522_N_254_O_126_	126	128	16,223.1

## Methods and Materials

### Synthesis of Polylysine Dendrimers

The polylysine dendrimers were synthesized by divergent synthesis as previously reported and obtained as their ammonium trifluoroacetate salts (one salt unit per amine group, Table 1).^39^ After deprotection, each dendrimer obtained was used as a starting material for the following generation. Using this method, it was possible to synthesize G1 to G6 of the PLL dendrimers with increasing molecular weights (MWs), which more than double from one generation to the next. This as well goes along with a consecutive increase in the number of lysine residues and surface NH_2_ groups (Table 1).

### Ion Mobility Mass Spectrometry

Dendrimer samples were analyzed in positive nanoelectrospray ionization (nESI) mode using an Agilent 6560 drift tube IM-Q-TOF instrument, a Waters Synapt HDS G2-Si traveling wave IM-MS instrument, and a home-built variable temperature IM-MS-QTof.^40^ A variety of solvents and solvent mixtures were tested to determine the most appropriate ones to use, including methanol, water, acetonitrile, and combinations of organic solvents with water in different ratios. Formic acid was added to promote protonation of the neutral dendrimers, where appropriate. All chemicals and solvents were obtained from Sigma-Aldrich. Following this optimization, dendrimer solutions were prepared in either methanol, pure water, or mixtures of water and methanol at concentrations between 1 and 20 μM, depending on the sensitivity of the instrument employed. To examine the entire family, a mix of all six generations was made (G1–G6) in water at a concentration of 2 μM per generation. These solutions were used to fill borosilicate capillaries (World Precision Instruments, Stevenage, U.K.), home-pulled from a Flaming/Brown P-2000 laser puller (Sutter Instrument Company, Novato, CA). In order to apply a voltage to the solution, a platinum wire (Diameter 0.125 mm, Goodfellow, Huntingdon, U.K.) was inserted, and capillary voltages between 1.0 and 1.4 kV were typically used. Typical instrument parameters for each instrument are found in the Supporting Information (Tables S1–S3).

### Activated IM-MS Experiments

On the Agilent 6560 instrument, activation was imparted by increasing the fragmentor voltage located within the source, which is located on top of the high-pressure funnel delta voltage. IM-MS data was acquired across the range of fragmentor voltages that allowed for the detection of dendrimer ions, from 400 to 600 V, and the latter is the upper boundary voltage that the instrument can apply. Below 400 V, dendrimer ions were not transmitted effectively. The fragmentor voltage was set at discrete values in increments of 50 V.

### Variable Temperature IM-MS Experiments

High-temperature data was collected using a variable temperature linear drift field ion mobility mass spectrometer (VT-IM-MS), previously reported.^40^ The drift cell can be operated over a pressure range of 0.5–3 Torr and temperatures between 150 and 520 K, with applied fields typically between 3 and 14 V cm^–1^. More details can be found elsewhere,^40^ and the parameters used for this work are presented in Table S3.

### Obtaining CCS Values from IM-MS Data

For data obtained using the Agilent 6560, CCS values were directly calculated with the stepped-field method, which does not require external calibration.^41^ For data from the traveling wave ion mobility capabilities of the Synapt G2-Si, CCS values were obtained using Agilent Tunemix^42^ and polyalanine as calibrants as previously described.^12,14^ We have applied a power law-based calibration method^43^ and a “blend + Radial” method^44^ to obtain CCS_N_2__ values for the dendrimers, and we have also then applied these calibrant CCS values to obtain the CCS value of Ubiquitin (see Supporting Information, Tables S6 and S8 for further details). For our home-built variable temperature IM-MS data,^40^ stepped-field IM-MS measurements in helium enabled the determination of dead time *t*_0_ and reduced mobility *K*_0_ necessary for the conversion of arrival time distributions (ATDs) into collision cross-section distributions (CCSDs), as described previously.^40^

## Results and Discussion

### Ion Mobility Mass Spectrometry Analysis of PLL Dendrimers

**Figure 2:**
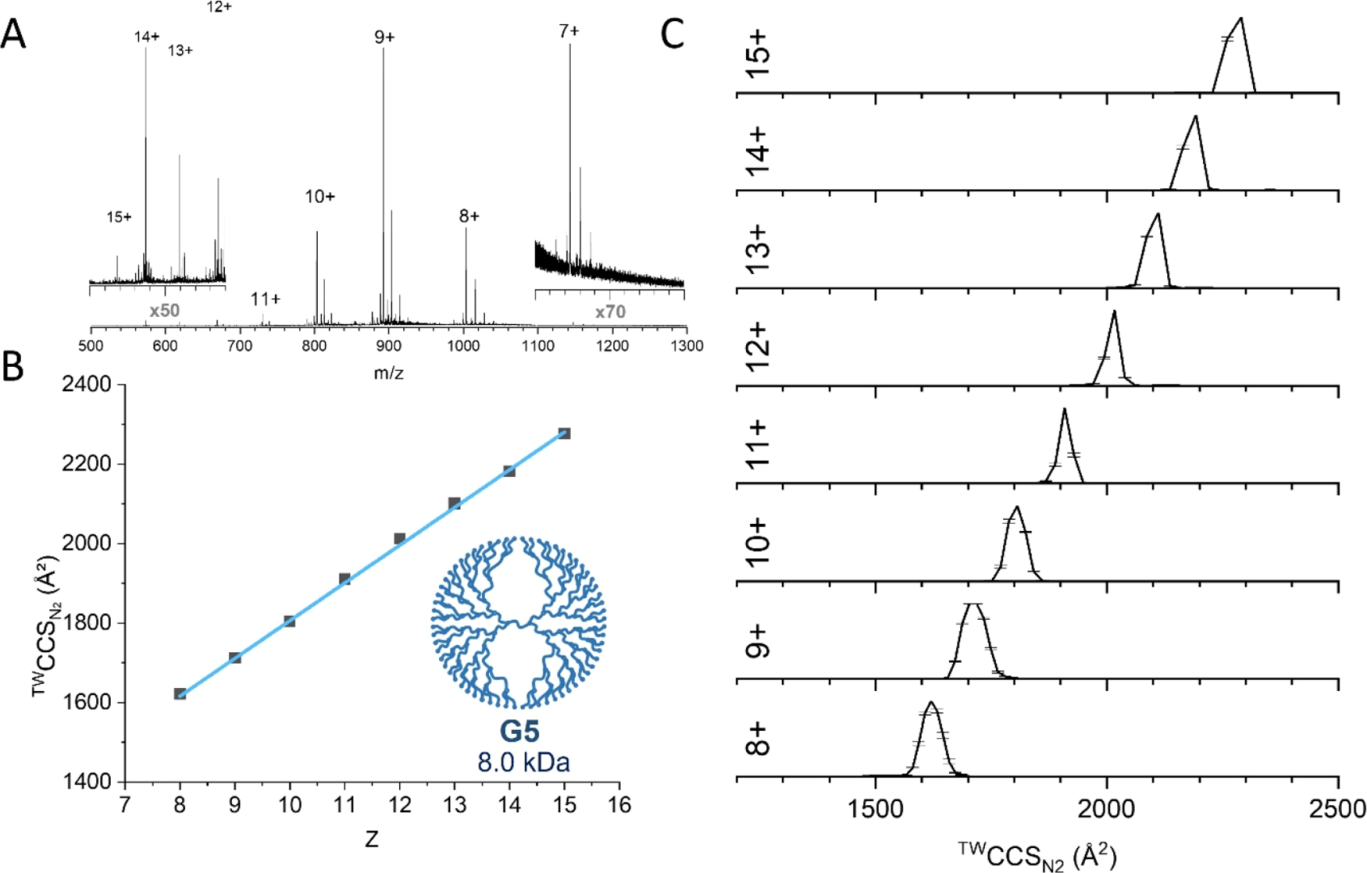
IM-MS data for the G5 PLL dendrimer and 1 μM aqueous solution acquired on Synapt G2-Si. (A) Mass spectrum of G5 PLL with the charge states labeled. (B) ^TW^CCS_N_2__ values (linear fit function: ^TW^CCS_N_2__ = 95.049*z* + 855.194, *R*^2^ = 0.999), and (C) ^TW^CCS_N_2__ distributions for a range of charge states. fwhm shown in Supporting Information, Table S9.

Mass spectra were obtained for all PLL dendrimer generations and a dendrimer mix (Figure S1) and showed high agreement between predicted monoisotopic and measured monoisotopic mass of the corresponding protomers across a range of charge states, where all ammonium trifluoroacetate salts were absent (Table S4). We chose the dendrimer G5 as an example to investigate dendrimer properties further with IM-MS. The mass spectra show a broad charge state distribution, ranging from 7+ ≤ *z* ≤ 15+ (Figure 2A), with salt adducts present, similar to the mass spectra of native proteins. These salts are likely bound to the terminal amino groups of the dendrimers.

Corresponding mass spectra from the dendrimer mix are shown in Figure S1, and the isolated earlier-generation dendrimers (G1–G3) are shown in Figure S2. The earlier-generation dendrimers presented some impurities and also showed higher in-source fragmentation or degradation species than G4–G6. Except for the G1 species, each polymer presents in higher-order charge states (Table S5).

Ion mobility data was obtained for each of the G5 charge states, and the corresponding CCS values were determined using TWIMS in nitrogen (^TW^CCS_N_2__, Figure 2B with charge states ranging from 7+ to 16+, Table S6). These show a strong linear trend with a monotonic increase in the CCS as a function of charge state and a CCS “gain” of approximately 95 Å^2^ per charge, with no shelving or leveling off. This data also shows a CCS increase of around 60% from 1502 to 2361 Å^2^ across the charge state range, which is remarkable since this is seemingly only imparted by the addition of protons. This behavior is similar to proteins and suggests that the synthetic architecture of the dendrimer (Figure 1) is forced to adopt discrete conformations as each proton is added that must overcome the spatial restriction of these synthesized compounds, which may be due to strong Coulombic repulsion. Changes in CCS with charge state have been previously reported for other dendritic systems. Saintmont et al. showed that for low charge states of PAMAM dendrimers, the CCS was similar (shelved), whereas for higher charge states, they observed a more significant increase in CCS similar to that observed with many proteins. For all of the charge states of the G5 PLL, the ^TW^CCS_N_2__ distributions obtained are unimodal (Figure 2C), suggesting that each charge state conformer adopts a single conformation in the gas phase. The full width at half-maximum (fwhm) values vary significantly between the charge states, interestingly showing a minimum for +11 and +12.

^DT^CCS_N_2__ values were obtained on the Agilent 6560 for this and all of the other PLL dendrimers (Table S5), and similar trends were observed. In each case, the CCS increases monotonically, and remarkably for the G4 PLL, the slope of the linear regression line (46 Å^2^ per charge) is lower than that for the G5 (81 Å^2^ per charge) and G6 (77 Å^2^ per charge, Figure S3). This may be due to the denser core of the two larger dendrimer generations, G5 and G6, with respect to G4. This is similar to the findings of Maire et al., who reported a monotonic increase in the CCS for a G3 PAMAM dendrimer with six to ten charges, where CCS values ranged from 917 to 1254 Å^2^.^45^ Interestingly, and in contrast to the data for the PLL dendrimers reported here, they observed that the CCS was independent of the charge state for low charge states of the lower PAMAM dendrimer generations. The authors hypothesized that starting from compact conformations, the dendrimers would expand to minimize Coulombic repulsion, and we suggest that this is also the case for the PLL dendrimers studied here (Figure 2 and Table S5).

### Extension to a Dendrimer Mix

To explore how these compounds could be used as IM-MS calibrants and as a system suitability test, we made and analyzed a dendrimer mix with a total of 22 ions across all six dendrimer generations (G1 – G6). Using the Agilent 6560 IM-QToF, the charge states sampled range from 1+ to 15+, and the corresponding ^DT^CCS_N_2__ values range from 182 to 2941 Å^2^ (Table S5). All CCS values measured were within 1% of one another over triplicate measurements, suggesting high reproducibility on the same instrument platform. While the arrival time distribution for each charge state of every dendrimer is narrow, with no indication of more than one conformer, remarkably there is a monotonic increase in ^DT^CCS_N_2__ with charge. This is in contrast to what is commonly observed for proteins of a similar size, where while there is a slight increase in CCS with increased *z*, often it is evident that conformations overlap charge states. This observation is explored in more detail below.

**Figure 3:**
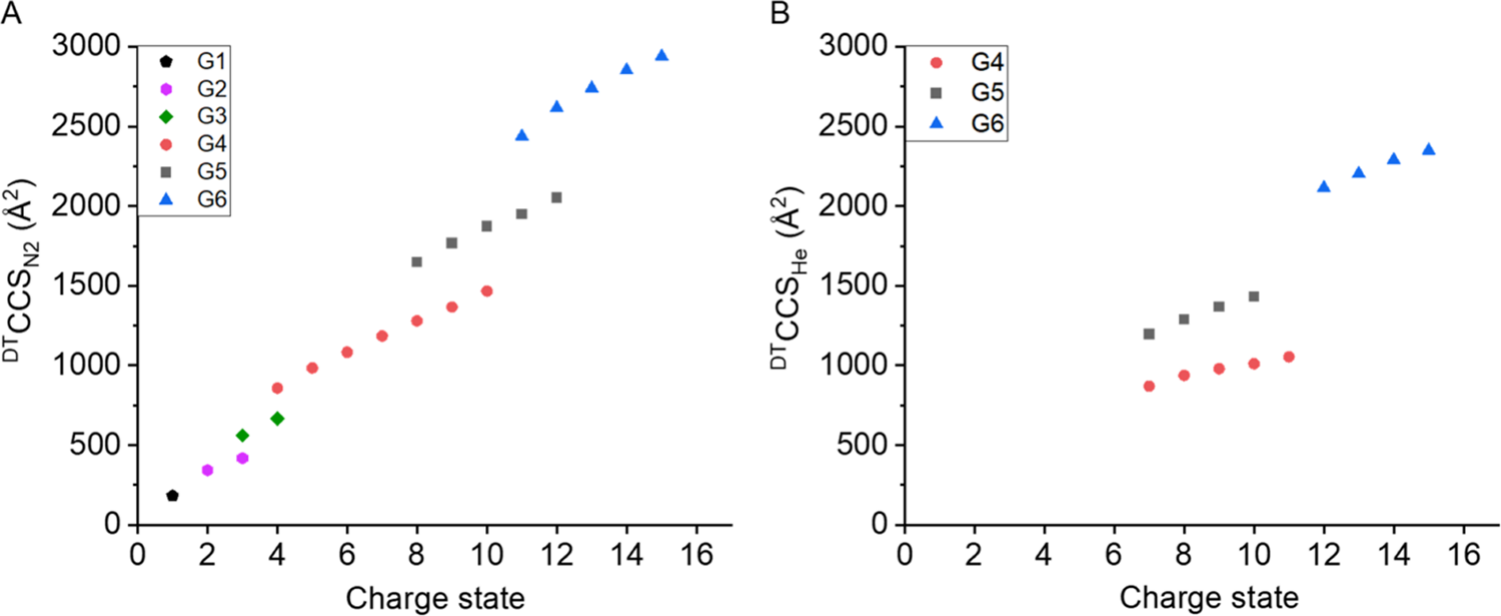
Comparison between ^DT^CCS_N_2__ and ^DT^CCS_He_, acquired from an Agilent 6560 IM-QToF. (A) ^DT^CCS_N_2__ trends as a function of charge for G1–G6 dendrimers, (B) ^DT^CCS_He_ trends as a function of charge G4, G5, and G6 dendrimers. ^DT^CCS_He_ values for G1–G3 are not presented due to low ion transmission. Solutions used were 2 μM G4/G5/G6 in water. Corresponding linear fit functions and *R*^2^ values are listed in Supporting Information, Table S10.

The ^DT^CCS_He_ values of the G4, G5, and G6 PLL dendrimers were also obtained using the Agilent 6560 IM-QToF platform (Figure 3b and Table S5). As with nitrogen, there is a linear increase in the CCS values as the charge increases for the three different samples present in different charge states.

### Collision-Induced Activation Experiments

**Figure 4:**
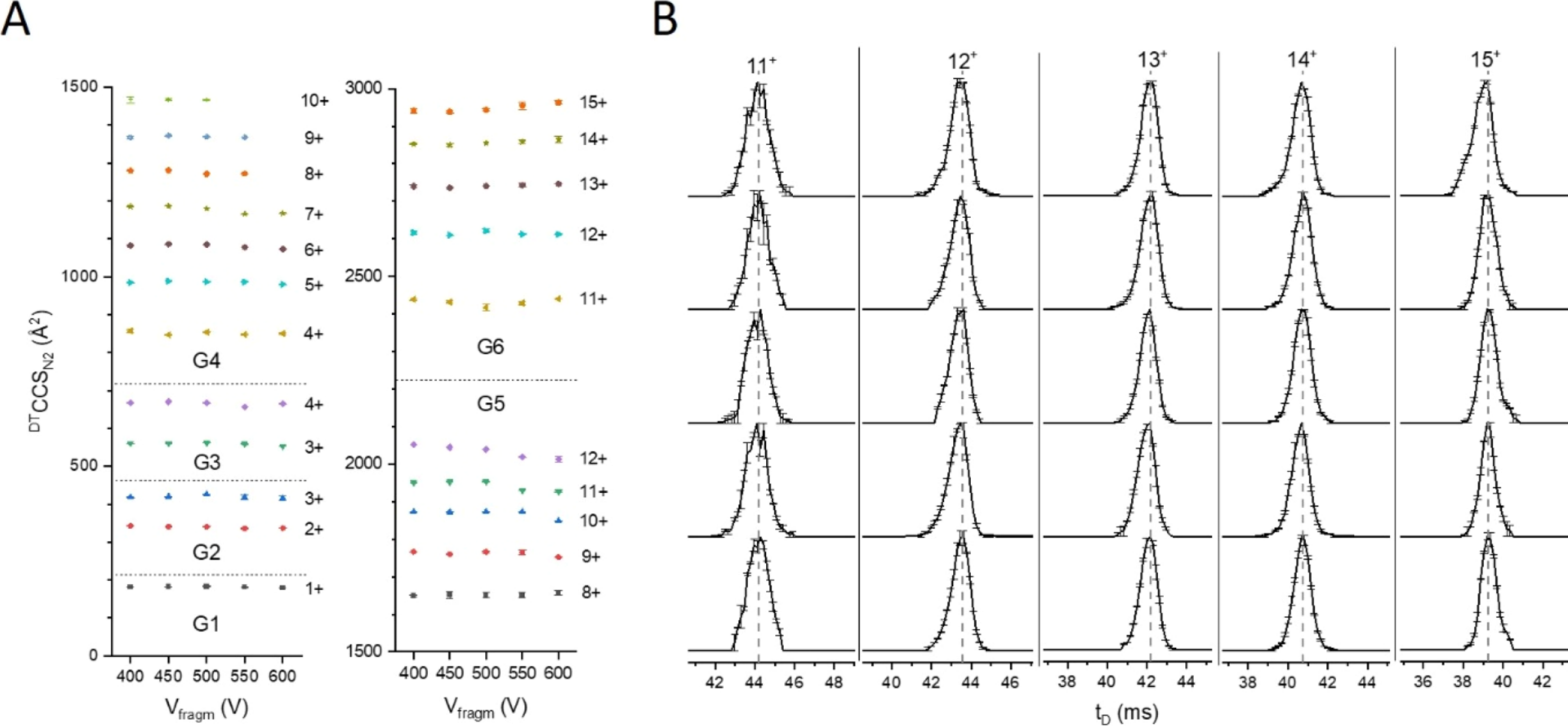
(A) In-source activation of the mixture of dendrimers (G1–G6) using the Agilent 6560 instrument with triplicate CCS values plotted as a function of in-source fragmentation voltage ranging from 400 to 600 V, using nitrogen buffer gas. We found little variation in the ^DT^CCS_N_2__ values with respect to increasing the fragmentor voltage (maximum 0.7%). (B) Arrival time distributions of the 11+, 12+, 13+, 14+, and 15+ G6 PLL dendrimer charge states obtained for increasing activation voltages ranging from 400 to 600 V acquired using the Agilent 6560 instrument in nitrogen. The vertical dashed lines represent the apex value of the arrival time distribution at the lowest activation voltage (400 V) for each charge state. For each dendrimer, stock solutions were prepared at 20 μM in water and diluted further to 1, 2, or 5 μM. The diluted mixture of the six dendrimers was prepared using the six stock solutions with a final concentration of 2 μM for each generation of dendrimer present.

In order to explore the stability of the dendrimers of different generations, the dendrimer mixture was subjected to in-source activation, followed by IM-MS measurement using the Agilent 6560 IM-QToF instrument. The measured ^DT^CCS_N_2__ values of each observed ion as a function of activation energy are shown in Figure 4 and Table S7. Remarkably, for every charge state, there is very little change in the ^DT^CCS_N_2__ values as a function of fragmentor voltage (Figure 4A); however, some variation was observed for the peak shape of the corresponding ATDs (Figure 4B). This indicates that the conformations discussed above are locked in by net charge, are robust, and cannot be readily distorted in great contrast to the behavior of linear polymers including proteins. Remarkably, these conformers are separable by IM alone (Figures S4–S6), suggesting an additional capability as IM calibrants.

Overall, all charge states of all generations show no or almost no CCS change, indicating high structural rigidity, whereas significant conformational transitions occur for comparable proteins such as native ubiquitin under similar activating conditions (Figure S7). This is particularly the case for G1–G3 and for the lower charge states of G4–G6, whereas the larger dendrimers in higher charge states exhibit slight conformational shifts. For G4, the higher charge states (8+, 9+, and 10+) no longer transmit at fragmentor voltages of 550 and 600 V. The 7+ charge state of G4 interestingly shows a slight decrease in CCS above 500 V fragmentor voltage (<2%). Similarly, the highest charge states of the G5 dendrimer (11+ and 12+) present a slight decrease in size (<2%) from 400 to 600 V.

### Variable Temperature Ion Mobility Mass Spectrometry Experiments

**Figure 5:**
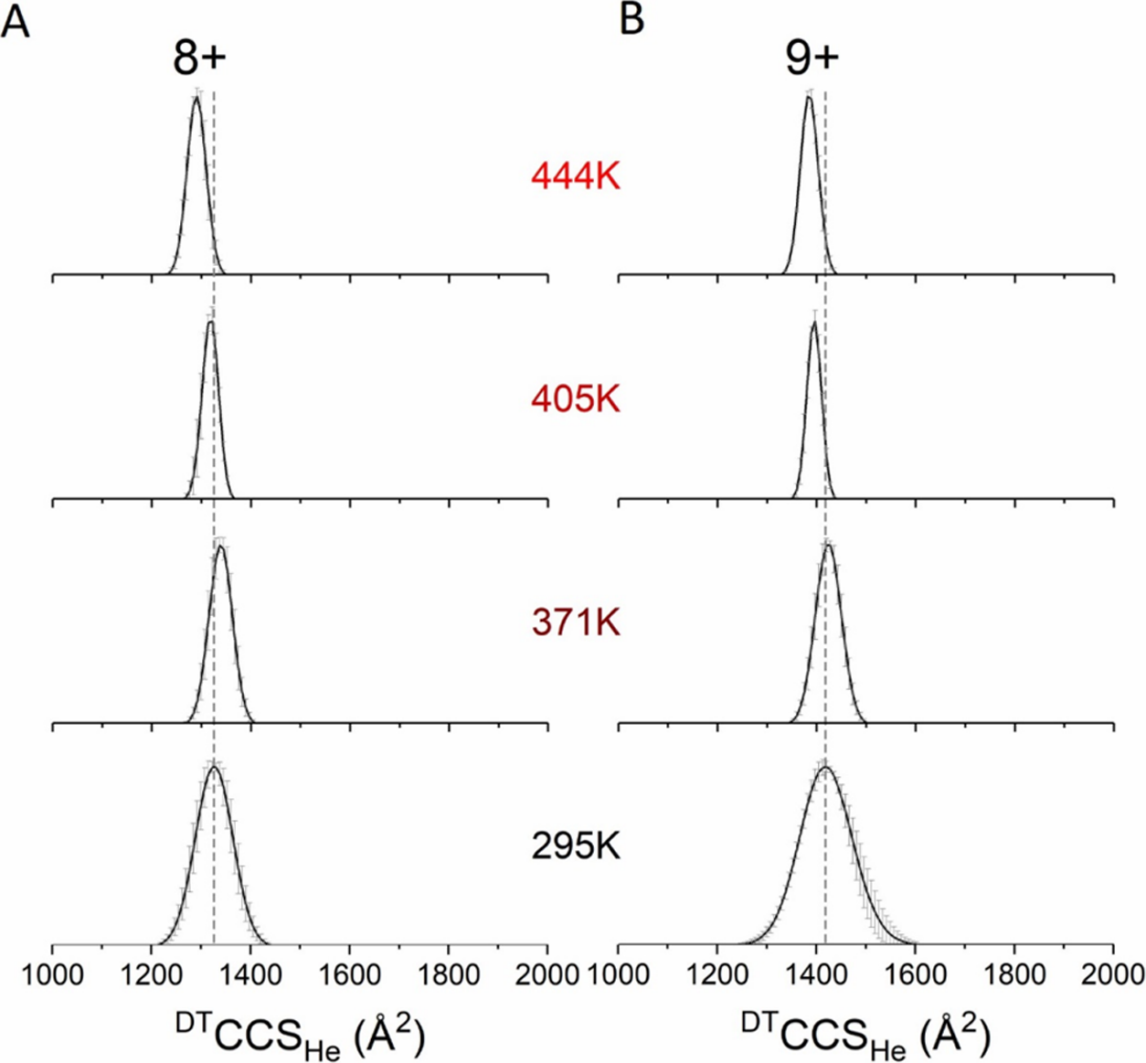
^DT^CCSD_He_ of (A) 8+ and (B) 9+ charge states of the G5 PLL dendrimer for helium buffer gas temperatures between *T* = 295 and 444 K. Data was acquired in triplicate using the home-built VT-IM-MS instrument and averaged. The gray, dashed lines show the apex ^DT^CCS_He_ value at room temperature, providing evidence for the minor unfolding from 295 to 371 K and the expected decrease in CCS as *T* increases due to more hard sphere-like interactions. Both G4 and G5 were prepared 20 μM in water.

Commercial ion mobility instrumentation often operates above the low field limit, which can lead to substantial activation and internal heating, as previously shown for both TWIMS^46,47^ and TIMS.^48^ Hence, CCS calibrants should exhibit strong rigidity and stability with respect to temperature, and for the 8+ and 9+ charge states of the G5 dendrimer, we explored this with our home-built variable temperature IM-MS instrument^40^ (Figure 5). While both charge states present unimodal distributions centered around ^DT^CCS_He_ = 1326 and 1419 Å^2^ at room temperature (295 K), respectively, consistent with the data from the Agilent 6560 IM-QToF (Table S5), the CCSDs at higher temperatures shift toward slightly lower CCS values with narrower peak widths. From room temperature to 444 K, the mean ^DT^CCS_He_ of the 8+ charge state decreases by 2.7% to 1291 ± 5 Å^2^, while the 9+ charge state experiences a 2.4% decrease to 1385 ± 1 Å^2^. Such trends in the CCS with higher temperatures are consistent with observations from Mason–Schamp,^49^ who suggested that the long-range attractive potential becomes less important at higher temperatures, as the collisions with the drift gas are more “hard sphere” like. Previous measurements on the Agilent TuneMix performed by us confirm this trend.^40^ The narrowing of the CCS distributions is less predicted and may indicate some annealing of the structures to discrete protomers at higher drift gas temperatures, as we have previously observed for proteins and protein complexes.^50,51^

### Structural Trends across the PLL Dendrimer Generations Compared to Proteins

**Figure 6:**
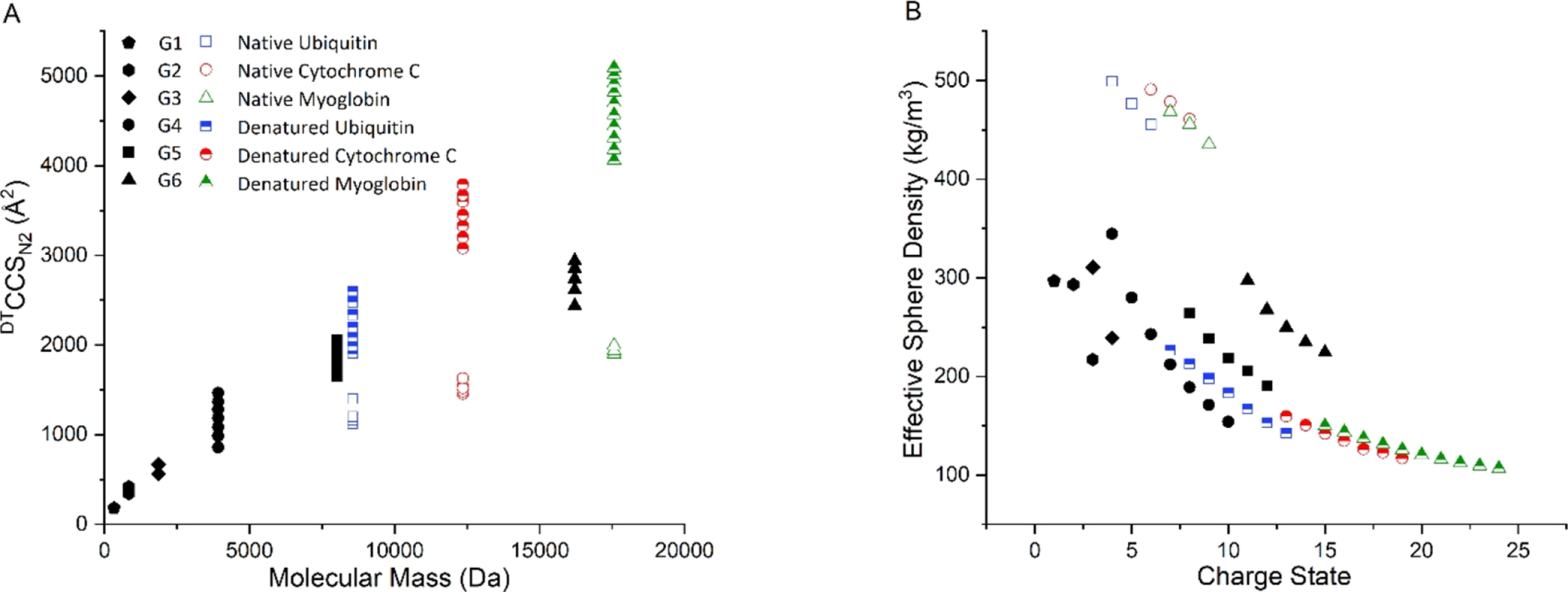
(A) ^DT^CCS_N_2__ range of the PLL dendrimer samples and standard proteins^12,15^ as a function of molecular mass, showing the CCS/mass trends according to density and ability to unfold in the gas phase. (B) Relationship between the effective sphere density (ESD) and charge state for ^DT^CCS_N_2__ of G1–G6 and the main conformer of selected native and denatured proteins. ESD was calculated from ^DT^CCS_N_2__ values based on the assumption of a spherical ion as previously suggested.^55^

The IM-MS data presented above revealed some remarkable trends. While the protonated mass spectra are highly similar to those of proteins, the ion mobility data from all instruments shows that these compounds are present as rigid conformers whose structures are imparted by charge and that these are very hard to deform in the mass spectrometer via collisional activation or heating of the drift gas. The differences in the range of CCS values found from these synthetic compounds compared to proteins of a similar molecular weight are shown in Figure 6. This plot is informative; it shows how linear polymeric proteins (namely ubiquitin, cytochrome C, and myoglobin) in their denatured state can all adopt a wider distribution of CCS values than the conformationally restricted dendrimers. The changes in CCS values of dendrimers may be better compared to those found for proteins like BTPI^52,53^ and lysozyme,^54^ which are conformationally restricted by disulfide bridges.

The correlation above between CCS and mass can inform on the packing density (“effective sphere density” = ESD) of a given ion, which is lowest for ions with high CCS and small mass. Introduced by our group for metalloproteins^55^ and later extended to intrinsically disordered proteins^56^ and metallosupramolecular complexes,^57^ these plots provide significant insights into the topology of gaseous ions and can aid in distinguishing compound families. In another work, we compared the packing density of the dendrimers with a range of other synthetic molecules. This showed one of the lowest packing densities of the nonbiological molecules studied and equally the highest CCS/packing density dependence on the charge state *z.*(58)

We calculated the ESD for every charge state of all six dendrimer generations (Figure 6B), showing a significantly decreased density for higher charge states within ions of the same dendrimer generation. This is in agreement with Coulombic repulsion similar to that of proteins, as discussed above. The data also shows that the higher dendrimer generations exhibit larger densities for ions with the same charge states, which agree with the structure of the dendrimers (Figure 1b).

The lower effective density of the dendrimers than proteins of equivalent mass is also evident from the charge state distributions. de la Mora provided an empirical relationship with which to predict a transition from a globular to a compact protein form based on the limit to the number of charges that a given protein can support as a globular species under ESI conditions (*z*_R_).^59^ This data is presented for the PLL dendrimer family in Table 2, along with the experimental data from charge state distributions, CCS distributions, and their variance in ^DT^CCS_N_2__, upon collisional activation, and effective sphere density range across different charge state states. *z*_R_ values are calculated for PLL dendrimers ranging from G1 to G6 and are compared to the *z*_min_ and Δ*z* values found experimentally.

**Table 2:** Summary of IM-MS Data for the PLL Dendrimers of G1–G6 with *z*_R_ (de la Mora Limit as Discussed above)^59^^a^^55^

generation	predicted monoisotopic mass (Da)	*z*_R_	*z*_min_	Δ*z*	Δ^DT^CCS_N2_ (Å^2^)	Δ^DT^CCS_N2_ CA (Å^2^)	Δρ (%)
G1	330.3	2	1	1		2.4	
G2	842.7	3	2	2	75.9	5.9	35.0
G3	1868.4	4	3	2	106.8	7.5	29.9
G4	3918.9	5	4	8	609.6	17.0	123.8
G5	8020.0	7	7	8	714.3	29.0	44.0
G6	16,223.1	10	11	5	502.0	22.0	32.4

a *z*_min_ as the minimum charge state; Δ*z* as the maximum difference between the highest and lowest charge state; Δ^DT^CCS_N_2__ and Δ^DT^CCS_N_2__ CA as the ^DT^CCS_N_2__ range upon collisional activation (Table S4–S6); and Δρ is the percentage difference in ESD between the most and least dense charge state, calculated based on ^DT^CCS_N_2__ values.^55^

From G1 to G4, the charge difference in the de la Mora limit for consecutive dendrimers is one, despite the fact that the mass of the dendrimers almost doubles from one generation to the next. This difference increases to two and three charges for G5 and G6 compared to their respective preceding generations, G4 and G5. Thus, for the G5 dendrimer, the de la Mora limit of *z*_R_ = 7 infers that charge states greater than or equal to 7+ correspond to somewhat extended forms. As shown in Figure 2, the charge states observed range from 7+ to 15+, indicating that all these conformers would present in rather extended structures in spite of the spatial restriction that the dendrimer architecture imposes. Similarly, the mass spectra of the G4 and G6 dendrimers show charge states that are higher than the respective *z*_R_ values (Table 2).

## Conclusions

We conclude that the studied poly(l-lysine) dendrimers are highly suitable as calibrants for ion mobility mass spectrometry. They are easy to synthesize, and their CCS values show a high reproducibility on and comparability between three different instrument platforms with two drift gases (helium and nitrogen). All of the ions exhibit unimodal CCS distributions, and across the six generations, we observed broad mass (*m* = 330–16,224 Da), charge state (*z* = 1–16), and CCS ranges (^DT^CCS_N_2__ = 182–2941 Å^2^), covering the calibration range necessary for many biomacromolecules and synthetic assemblies. We probed the structural stability of these polymers upon collisional and thermal activation, showing only minor CCS changes in both cases. This apparent rigidity of the dendrimers is in contrast to the flexibility of proteins in the gas phase, making the dendrimers, in combination with the other advantages listed above, potentially more suitable for CCS calibration than native or denatured proteins. We have also applied both power law^43^ and blend function^44^ methods of calibration to obtain the CCS values from TWIMS data and find small differences and slightly closer calibrated values to experiment with the blend function method (Supporting Information, Table S8), which may be useful in the future evaluation of calibration methods and indeed in the use of polylysine dendrimers as calibrants.

Our results also provide insights into the effect of multiple charges on spatially restricted molecules such as the studied dendrimers. We found that Coulombic repulsion forces govern significant extensions and decrease in packing density at higher charge states (Figure 6), which is well-known for three-dimensional biomacromolecules but so far unusual for two-dimensional synthetic architectures.

## Supporting Information Available

Detailed typical instrument parameters for the Synapt G2-Si; the Agilent 6560 IM-QToF, and also for the variable temperature IM-MS instrument; predicted and measured monoisotopic masses for the G1–G6 family of dendrimers and provide mass spectra for the dendrimer mix and for each of G1, G2, and G3; the ^DT^CCS values for all observed charge states of all dendrimers examined on each instrument, as well as CCS_He_ and CCS_N_2__ values for the G5 dendrimer charge states, and ^DT^CCS_N_2__ apex values and CCS distributions with in-source fragmentation voltages ranging from 400 to 600 V (PDF)
