# Supplementary material for: Exploring the Conformational Landscape of Poly(l-lysine) Dendrimers Using Ion Mobility Mass Spectrometry
Source: Anal Chem. 2024 May 30;96(23):9390–8. doi: 10.1021/acs.analchem.4c00099 (PMC11170554; doi:10.1021/acs.analchem.4c00099)
Supplement: Supplementary file 1 — ac4c00099_si_001.pdf [file ac4c00099_si_001.pdf]

Supplementary Information for

Exploring the Conformational Landscape of Poly(L-lysine) Dendrimers using Ion Mobility Mass Spectrometry

Florian Benoit[1, #], Xudong Wang[1, #], Junxiao Dai[1], Niklas Geue[1], Richard M. England[2], Anthony W. T. Bristow[2] and Perdita E. Barran[1,*]

[1]Michael Barber Centre for Collaborative Mass Spectrometry, Manchester Institute of Biotechnology, Department of Chemistry, The University of Manchester, 131 Princess Street, Manchester, M1 7DN, UK. [2]Chemical Development, Pharmaceutical Technology and Development, Operations, AstraZeneca, Charter Way, Macclesfield, SK 102NA, UK.

[*]Corresponding Author: perdita.barran@manchester.ac.uk

[#]These two authors contributed equally to this manuscript.

Table of Contents

Table S1. Typical instrument parameters used on the Synapt G2-Si platform. 4

Table S2. Typical instrument parameters used on the Agilent 6560 IM-QToF platform. 5

Table S3. Typical instrument parameters used on the home-built variable temperature IM-MS platform. 6

Figure S1. Mass spectra of the mixture of six dendrimers G1 to G6 7

Table S4. Predicted and measured monoisotopic masses acquired of the dendrimer mix 8

Figure S2. Mass spectra of (A) 20 µM G1 PLL in methanol, (B) 10 µM G2 PLL in methanol and (C) 20 µM G3 PLL dendrimer in methanol-water 1:1 (v/v), 0.1 % FA acquired on the Synapt G2-S in nESI positive ionisation mode using settings as shown in Table S1. 10

Table S5. ^{DT}CCS values measured using the Agilent 6560 IM-QTOF platform in nitrogen and helium using the instrument settings described in Table S2. $^{DT}CCS_{He}$ values were measured for individual dendrimers, whereas $^{DT}CCS_{N2}$ values were mostly acquired for the dendrimer mix (2 µM in water). The effective spherical density (ESD) was calculated based on the DTCCSN2 values and (see caption of Figure 6). 12

Table S6. Comparison of CCS_{He} and CCS_{N2} values of the G5 dendrimer between three different instrument platforms. where $^{DT}CCS_{N2}$ is measured individually and exhibits a wider CSD than in the dendrimer mix. 13

Figure S3. ^{DT}CCS values as a function of charge state measured on the Agilent 6560 IM-QTOF (5 µM in water). (A) G4 dendrimer G4-He: $^{DT}CCS_{He}$ = 44.3z + 572.0, R^2 = 0.980. G4-N2: $^{DT}CCS_{N2}$ = 101.9z + 454.0, R^2 = 0.996.(B) the G5 dendrimer G5-He: $^{DT}CCS_{He}$ = 78.2z + 656.2, R^2 = 0.993. G5-N2: $^{DT}CCS_{N2}$ = 101.9z + 454.0, R^2 = 0.996. 14

Table S7. $^{DT}CCS_{N2}$ values of the mixture of dendrimers 15

Figure S4. Arrival time distributions (ATDs) of the (A) charge state 1+ of G1, (B) charge state 2+ of G2, (C) charge state 3+ of G2, (D) charge state +3 of G3 and (E) charge state +4 of G3............................ 16

Figure S5. Arrival time distributions (ATDs) of dendrimer G4 between the charge states 4+ (A) and 9+ (F). ATDs were obtained for increasing activation voltages ranging from 400 to 600 V acquired on the Agilent 6560 IM-QToF in nitrogen 17

Figure S6. Arrival time distributions (ATDs) of dendrimer G5 between the charge states 8+ (A) and 12+ (F). ATDs were obtained for increasing activation voltages ranging from 400 to 600 V acquired on the Agilent 6560 IM-QToF in nitrogen 18

Figure S7. Arrival time distributions (ATDs) of the (A) 8+ and (B) 9+ native ubiquitin charge states obtained for increasing activation voltages ranging from 40 to 190 V 19

Table S8. Ubiquitin $^{TW}CCS_{N2}$ calibrated by G5-Dendrimers using the power law[3] and the 'Blend+Radial' methods[4] each compared with reference CCS_{N2} data from Bush *et al.*[5]. Ubiquitin data was acquired in 5µM in water/methanol/acetic acid (49/49/2) on a Synapt G2-Si. 20

Table S9. FWHM (Full width at half maximum) of Figure 2C, minimum for the 11+ and 12+ charge states indicate sub structure that is potentially more rigid/monodisperse for these two charge states. 20

Table S10. Fit function and R^2 value for $^{DT}CCS_{N2}$ and $^{DT}CCS_{He}$ from Figure 3........ 21

References 22

Source voltages (V) and gas flows (mL/min)	
Capillary voltage (kV)	1.1 – 1.5
Source temperature (°C)	80.0
Sample cone	20.0
Extractor cone	5.0
Trap gas flow	2.0
Helium cell gas flow	180
IMS gas flow	90.0
**DC voltages**	
Trap cell energy (V)	0.0
Trap DC entrance	0.0
Trap DC bias	45.0
Trap DC	0.0
Trap DC exit	3.0
Trap height (V)	2
IMS DC entrance	25.0
Helium cell DC	35.0
Helium cell exit	-5.0
IMS bias	3.0
IMS DC exit	0.0
Transfer cell energy (V)	0.0
Transfer DC entrance	4.0
Transfer DC exit	15.0
**RF voltages (V)**	
Source	350
Trap	300
IMS	300
IMS mobility	250
Transfer	350
**Wave velocities (ms^{-1}) and heights (V)**	
Source wave velocity	200
Source wave height	0.2
Trap wave velocity	313
Trap wave height	2.0
IMS wave velocity	650
IMS wave height	25.0
Transfer wave velocity	300
Transfer wave height	4.0
**Pressures (mbar)**	
Backing	2.71
Trap	$2.41e^{-2}$
Helium cell	$1.40e^{3}$
IM cell	3.56
Transfer	$2.59e^{-2}$
ToF	$1.06e^{-6}$

Table S1. Typical instrument parameters used on the Synapt G2-Si platform.

Source voltages (V) and gas flows (L/min)		
Capillary voltage (kV)	1.0 – 1.4	
Source temperature (°C)	50.0-80.0	
Drying gas flow	2	
Fragmentor	350-400	
**DC voltages**		
HP Funnel delta	120-180	
Trap entrance grid delta	1	
Trap entrance grid low	96	
Trap entrance grid high	106.0	
Trap entrance	91.1	
Trap exit	89.9	
Trap exit grid 1 delta	4	
Trap exit grid 1 low	89.1	
Trap exit grid 1 high	93.1	
Trap exit grid 2 delta	7.1	
Trap exit grid 2 low	87.5	
Trap exit grid 2 high	96.7	
Trap Funnel delta	177.1	
IM Hexapole delta	-8	
IM Hexapole entrance	41	
Rear Funnel entrance	240	
Rear Funnel exit	45	
**RF voltages (V)**		
Octapole	750	
High Pressure Funnel	100-140	
Trap Funnel	100-160	
IM Hexapole	300	
Rear Funnel	200	
**Other parameters**		
Trap fill time (µs)	5000	
Trap release time (µs)	150	
**Pressures (Torr)**		
	**He**	**N_2**
HP Funnel	4.50-4.80	4.30-4.70
Drift tube	3.89	3.90
Trap Funnel	3.76	3.62

Table S2. Typical instrument parameters used on the Agilent 6560 IM-QToF platform.

Source voltages (V)	
Capillary voltage (kV)	1.0 – 1.4
Source temperature (°C)	50.0
Desolvation temperature (°C)	120
Fragmentor	350-400
**DC&RF voltages (V)**	
LM Resolution	5.0
HM Resolution	5.0
Collision Energy	1.0
Ion Energy	1.0
Steering	0.00
Entrance	50.0
Pre-filter	5.0
Transport	5.0
Aperture2	5.0
Acceleration	200
Focus	0
Tube Lens	70
Offset1	0.3
Offset2	0.0
Pusher	980
TOF (kV)	9.10
Reflectron	33.50
Aperture2	5.0
Acceleration	200
TDC Start	1.25
TDC Stop	0.055
**Other parameters**	
Pusher Cycle Time (µs)	80
Pusher Frequency (Hz)	12500
**Pressures**	
Drift tube(bar)	2.05-2.10
Pirani Pressure(mbar)	7.20e-1
Penning Pressure(mbar)	6.13e-6
Tof Penning Pressure(mbar)	5.49e-7

Table S3. Typical instrument parameters used on the home-built variable temperature IM-MS platform.

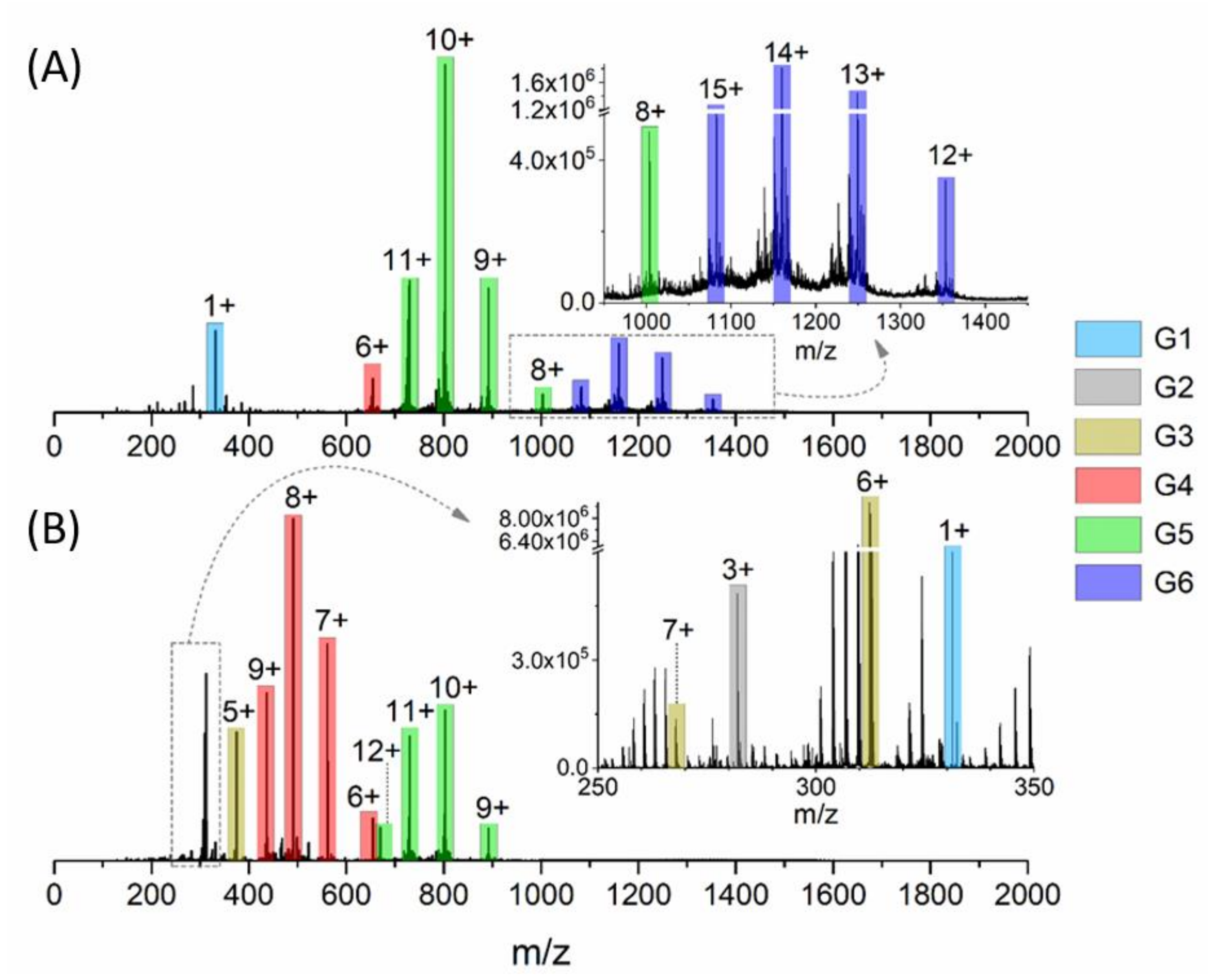

Figure S1. Mass spectra of the mixture of six dendrimers G1 to G6 (2 µM in water) acquired using the Synapt G2-Si platform in positive nESI mode with a cone voltage of (A) 80 V and (B) 20 V. The charge states of the G1 (cyan), G2 (grey), G3 (yellow), G4 (red), G5 (green) and G6 (dark blue) PLL dendrimers are indicated on both spectra. Inset regions are shown for the m/z ranges 950 – 1450 for the first one and 250 – 350 for the second one. Harsher source conditions (higher cone voltage) was shown to induce more efficient desolvation of higher generation dendrimers, and for a given dendrimer, harsher source conditions generate lower charge states.

Dendrimer	Predicted Monoisotopic Mass M of Neutral Dendrimer (Da)	Measured Mass M (Da) Agilent 6560	Measured Mass M (Da) Synapt G2	Maximum Δz
G1	330.3	330.3	330.3	1
G2	842.7	842.8 ± 0.1	842.7	2
G3	1868.4	1868.4 ± 0.1	1868.5 ± 0.1	2
G4	3918.9	3919.1 ± 0.1	3919.4 ± 0.3	8
G5	8020.0	8020.2 ± 0.4	8020.9 ± 0.6	8
G6	16223.1	16223.7 ± 0.5	16224.1 ± 0.8	5

Table S4. Predicted and measured monoisotopic masses acquired of the dendrimer mix (2 µM in water) from the Agilent 6560 Q-TOF/Synapt G2-Si in positive nESI mode. Measured monoisotopic mass was averaged from one measurement across different charge states, and no error could be obtained for G1 as only one charge state is present. The range of charges seen with nitrogen as the drift gas are indicated as Δz. When run as a mixture, tuning conditions to best optimise each resulted in Δz values that were lower compared to individual runs of the isolated dendrimers. Charge state ranges vary slightly across different instruments and drift gases, and we have noted the values that are largest across all acquired datasets and both drift gases. Errors were reported for those dendrimers that exhibit more than one charge state.

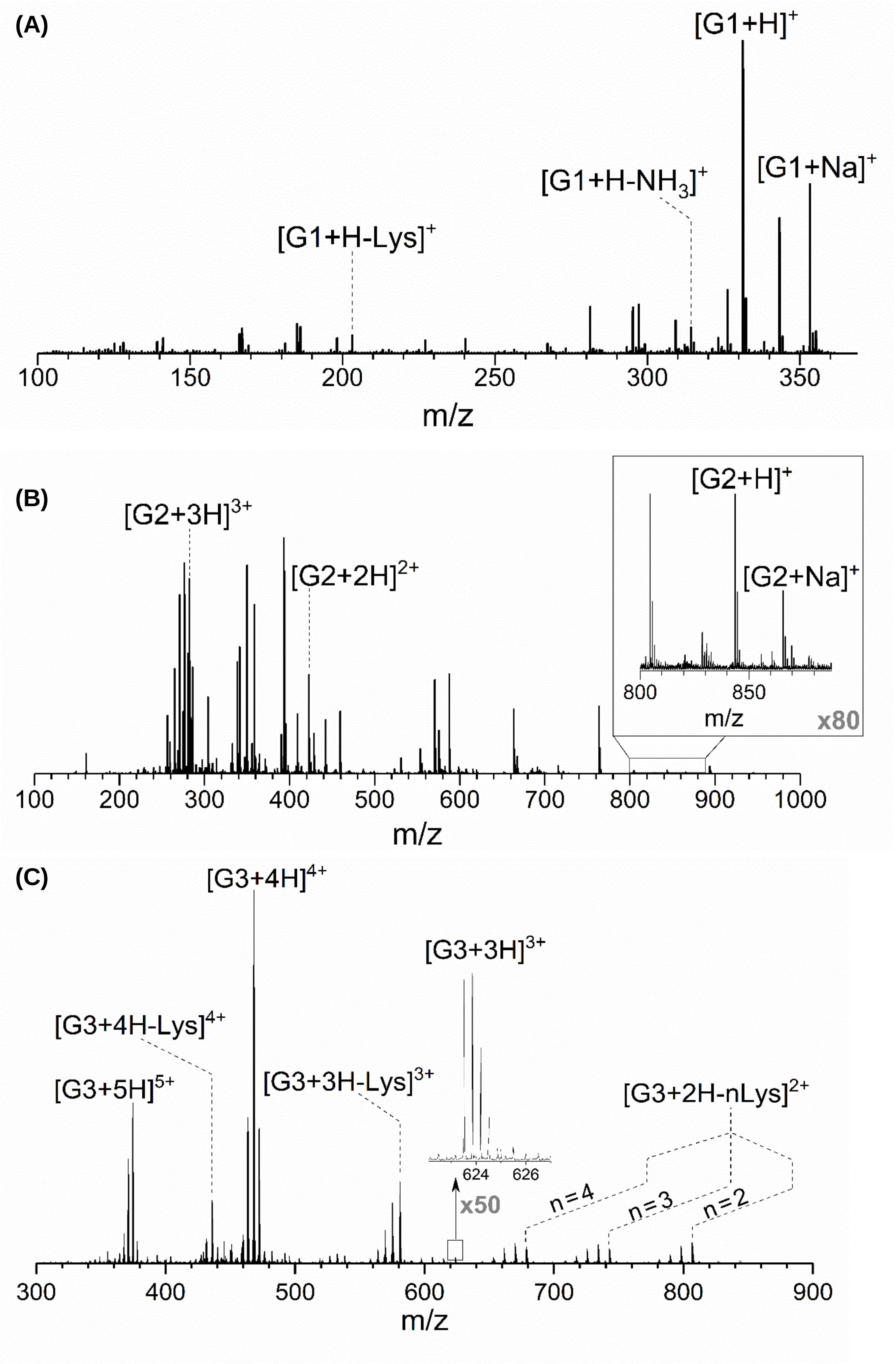

(A)
[G1+H]+
[G1+Na]+
[G1+H-NH3]+
[G1+H-Lys]+
100
150
200
250
300
350
m/z
(B)
[G2+3H]3+
[G2+2H]2+
[G2+H]+
[G2+Na]+
800
850
m/z
x80
100
200
300
400
500
600
700
800
900
1000
m/z
(C)
[G3+4H]4+
[G3+3H]3+
[G3+4H-Lys]4+
[G3+5H]5+
[G3+3H-Lys]3+
[G3+2H-nLys]2+
624
626
x50
n=4
n=3
n=2
300
400
500
600
700
800
900
m/z

Figure S2. Mass spectra of (A) 20 µM G1 PLL in methanol, (B) 10 µM G2 PLL in methanol and (C) 20 µM G3 PLL dendrimer in methanol-water 1:1 (v/v), 0.1 % FA acquired on the Synapt G2-S in nESI positive ionisation mode using settings as shown in Table S1.

The G1 dendrimer (M = 330 Da) only presents as singly charged ions, either in their protonated or sodiated form at m/z 331 or 353, respectively. Other features shown in the mass spectrum include ions that lost an amino group at m/z 314 or a lysine group at m/z 203. These ions could arise from by-products during the organic synthesis of the monodisperse fully branched dendrimer, in turn showing mass defects compared with the mass of the expected final product. Branching or mass defects were observed previously for other systems such as PAMAM dendrimers.[1]

The mass spectrum of the next generation dendrimer, G2 PLL, presents in three different charge states with low levels of singly protonated and sodiated ions at *m/z* 844 and 866, and a variety of doubly and triply protonated ions with respective precursor ions at *m/z* 422 and 282, as labelled in Figure S2b. The triply protonated ion $[G2+3H]^{3+}$ is accompanied by losses of $–NH_2$ groups and mass defects of 128 Da are also observed for the singly and doubly protonated ions (mass of a lysine residue). For example, the singly protonated G2 precursor ion shows a mass defect of one, two and three lysine residues at *m/z* 716, 588 and 459, respectively. Again, these can be attributed to by-products or subsequent fragmentation in the mass spectrometer under activating conditions. There are other unidentified peaks in the mass spectrum that may correspond to supplementary contaminants and by-products, possibly originating from the synthetic route chosen.

For the G3 PLL dendrimer mass spectra, shown in Figure S2c, the charge state distribution observed is broader than for the previous dendrimers analysed. The main charge states range from 3+ to 5+ centred on the 4+ for the fully branched G3 dendrimer. Interestingly, some mass defects can be observed with a loss of 128 Da indicative of a lysine residue, which is the case for the 3+ and 4+ charge states at *m/z* 581 and 436, respectively. However, for the 2+ charge state, a succession of two, three and four lysine residue mass defects can be observed at *m/z* 807, 743 and 679, respectively. With fewer lysine residues within the dendrimer architecture, the amount of charges that can be solvated decreases, which can explain the presence of these doubly protonated ions at higher *m/z* values, relative to other charge

states. In addition, all of the charge states present in the mass spectrum show degrees of –NH_2 amino loss or mass defect, with losses of up to four amino groups, as shown for the dendrimer ion $[G3+3H\text{-}Lys]^{3+}$ at *m/z* 581. This was shown to happen for protonated lysine residues with which the side chain amino group picks up a proton, and a subsequent cyclisation produces NH_3 with a loss of 17 Da. The NH_3 loss arising from the side chain has been demonstrated using ^{15}N-labelling.[2]

Dendrimer Generation	Charge z	m/z	$^{DT}CCS_{N2}$ (Å^2)	$^{DT}CCS_{He}$ (Å^2)	ESD (kg/m^3) based on $^{DT}CCS_{N2}$
**G1**	1	331.3	182.1 ± 0.1	-	296.8
**G2**	2	422.4	342.8 ± 1.0	-	293.2
	3	282.3	418.7 ± 0.4	-	217.2
**G3**	3	623.8	560.7 ± 0.3	-	310.6
	4	467.9	667.5 ± 0.5	-	239.1
**G4**	4	980.9	857.4 ± 4.8	-	344.6
	5	784.9	984.9 ± 0.4	-	279.9
	6	656.1	1083.0 ± 2.0	-	242.7
	7	560.9	1185.0 ± 1.0	869.7 ± 2.0	212.1
	8	490.9	1280.0 ± 1.0	937.8 ± 2.0	188.9
	9	436.5	1367.0 ± 3.0	979.5 ± 4.0	171.2
	10	392.9	1467.0 ± 8.0	1010.9 ± 2.0	154.0
	11	357.3	-	1054.5 ± 3.0	-
**G5**	7	1147.3	-	1196.0 ± 5.4	-
	8	1003.6	1655.4 ± 6.4	1289.3 ± 9.4	264.4
	9	892.1	1773.6 ± 6.6	1366.7 ± 8.6	238.4
	10	803.1	1877.8 ± 4.8	1430.7 ± 11.4	218.4
	11	730.2	1954.9 ± 3.9	-	205.5
	12	669.4	2041.3 ± 10.7	-	190.5
**G6**	11	1475.9	2439.0 ± 3.0	-	297.3
	12	1353.0	2617.0 ± 4.0	2115.4 ± 23.0	267.5
	13	1249.0	2740.0 ± 4.0	2205.0 ± 9.0	249.7
	14	1159.9	2853.0 ± 1.0	2291.5 ± 16.0	235.0
	15	1082.6	2941.0 ± 5.0	2349.4 ± 11.0	224.5

Table S5. ^{DT}CCS values measured using the Agilent 6560 IM-QTOF platform in nitrogen and helium using the instrument settings described in Table S2. $^{DT}CCS_{He}$ values were measured for individual dendrimers, whereas $^{DT}CCS_{N2}$ values were mostly acquired for the dendrimer mix (2 µM in water). The effective spherical density (ESD) was calculated based on the DTCCSN2 values and (see caption of Figure 6).

**Charge State**	**$^{DT}CCS_{He}$ (Å^2) (Agilent 6560)**	**$^{DT}CCS_{He}$ (Å^2) (VT-IM-MS)**	**$^{DT}CCS_{N2}$ (Å^2) (Agilent 6560)**	**$^{TW}CCS_{N2}$ -PL (Å^2) (Synapt G2s)**	**$^{TW}CCS_{N2}$ -BR(Å^2) (Synapt G2s)**
**7**	1196.0 ± 5.4	-	1512.2 ± 10.3	1502.1 ± 1.1	1522.3 ± 1.3
**8**	1289.3 ± 9.4	1316.0 ± 8.3	1655.4 ± 6.4	1621.3 ± 0.9	1631.9 ± 0.7
**9**	1366.7 ± 8.6	1402.1 ± 23.1	1773.6 ± 6.6	1712.8 ± 0.3	1707.4 ± 0.3
**10**	1430.7 ± 11.4	-	1877.8 ± 4.8	1804.0 ± 0.4	1804.6 ± 0.1
**11**	-	-	1954.9 ± 3.9	1910.4 ± 0.6	1898.4 ± 0.6
**12**	-	-	2041.3 ± 10.7	2012.2 ± 0.2	1997.0 ± 0.8
**13**	-	-	2131.3 ± 31.0	2101.3 ± 0.1	2081.9 ± 1.0
**14**	-	-	2226.5 ± 17.5	2182.4 ± 0.2	2155.8 ± 1.2
**15**	-	-	-	2277.3 ± 0.3	2243.0 ± 1.5
**16**	-	-	-	2361.4 ± 0.3	2483.2 ± 5.1

Table S6. Comparison of CCS_{He} and CCS_{N2} values of the G5 dendrimer between three different instrument platforms. where $^{DT}CCS_{N2}$ is measured individually and exhibits a wider CSD than in the dendrimer mix. TWCCSN2 -PL is obtained using a power law method3, TWCCSN2 -BR is obtained via a 'Blend+Radial' method as reported by Richardson et al4.

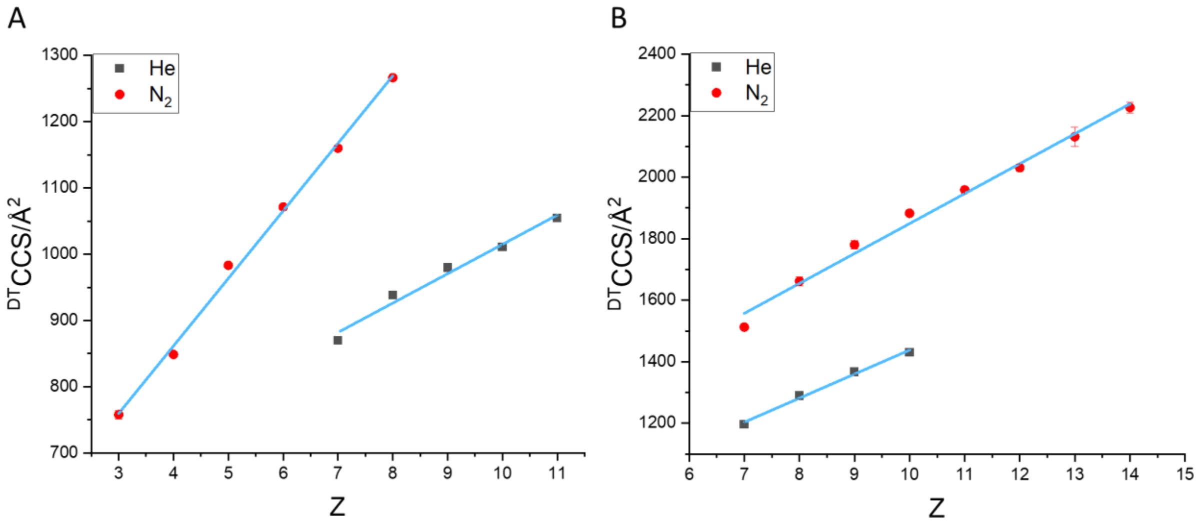

Figure S3. ^{DT}CCS values as a function of charge state measured on the Agilent 6560 IM-QTOF (5 µM in water). (A) G4 dendrimer G4-He: $^{DT}CCS_{He}$ = 44.3z + 572.0, R^2 = 0.980. G4-N2: $^{DT}CCS_{N2}$ = 101.9z + 454.0, R^2 = 0.996.(B) the G5 dendrimer G5-He: $^{DT}CCS_{He}$ = 78.2z + 656.2, R^2 = 0.993. G5-N2: $^{DT}CCS_{N2}$ = 101.9z + 454.0, R^2 = 0.996. The increase in CCS for G5 is quantitatively the same in each gas (Figure S3 B), for G4 it is half the magnitude in helium than in nitrogen (Figure S3 A), this is an unexpected and interesting observation, this suggests a different restructuring for each charge conformer occurs for this smaller dendrimer in each gas.

Generation	z	400 V	450 V	500 V	550 V	600 V
G1	1	182.1 ± 0.1	182.3 ± 0.2	182.6 ± 1.1	181.3 ± 0.5	179.7 ± 0.1
G2	2	342.8 ± 1.0	341.0 ± 2.0	340.4 ± 0.8	336.2 ± 1.2	336.9 ± 0.6
	3	418.7 ± 0.4	420.0 ± 0.5	426.0 ± 0.8	418.5 ± 6.0	416.3 ± 6.3
G3	3	560.7 ± 0.3	561.1 ± 0.5	561.5 ± 0.1	559.4 ± 1.1	553.2 ± 0.3
	4	667.5 ± 0.5	670.4 ± 0.1	667.9 ± 0.6	656.9 ± 0.8	665.8 ± 0.9
G4	4	857.4 ± 4.8	846.9 ± 0.9	853.7 ± 0.3	847.9 ± 2.3	850.5 ± 2.5
	5	984.9 ± 0.4	988.6 ± 1.0	986.7 ± 0.5	986.7 ± 0.5	980 ± 0.4
	6	1083 ± 2	1086 ± 1	1085 ± 1	1078 ± 1	1073 ± 1
	7	1185 ± 1	1186 ± 1	1180 ± 0	1166 ± 1	1168 ± 1
	8	1280 ± 1	1281 ± 1	1271 ± 1	1272 ± 1	
	9	1367 ± 3	1373 ± 2	1369 ± 2	1368 ± 1	
	10	1467 ± 8	1467 ± 2	1467 ± 1		
G5	8	1649 ± 2	1651 ± 10	1651 ± 8	1652 ± 7	1658 ± 6
	9	1767 ± 1	1760 ± 1	1766 ± 2	1765 ± 5	1752 ± 1
	10	1873 ± 2	1873 ± 1	1873 ± 1	1873 ± 2	1849 ± 1
	11	1951 ± 2	1953 ± 1	1954 ± 1	1930 ± 1	1927 ± 2
	12	2052 ± 1	2045 ± 1	2039 ± 1	2019 ± 2	2013 ± 6
G6	11	2439 ± 3	2432 ± 3	2417 ± 9	2429 ± 6	2440 ± 1
	12	2617 ± 4	2610 ± 1	2621 ± 5	2612 ± 1	2612 ± 1
	13	2740 ± 4	2736 ± 1	2741 ± 1	2743 ± 5	2746 ± 1
	14	2853 ± 1	2850 ± 3	2855 ± 0	2859 ± 5	2865 ± 9
	15	2941 ± 5	2939 ± 3	2944 ± 3	2955 ± 10	2963 ± 5

Table S7. $^{DT}CCS_{N2}$ values of the mixture of dendrimers (G1 to G6, 2 µM in water for each dendrimer) measured in duplicate with in-source fragmentation voltages ranging from 400 to 600 V.

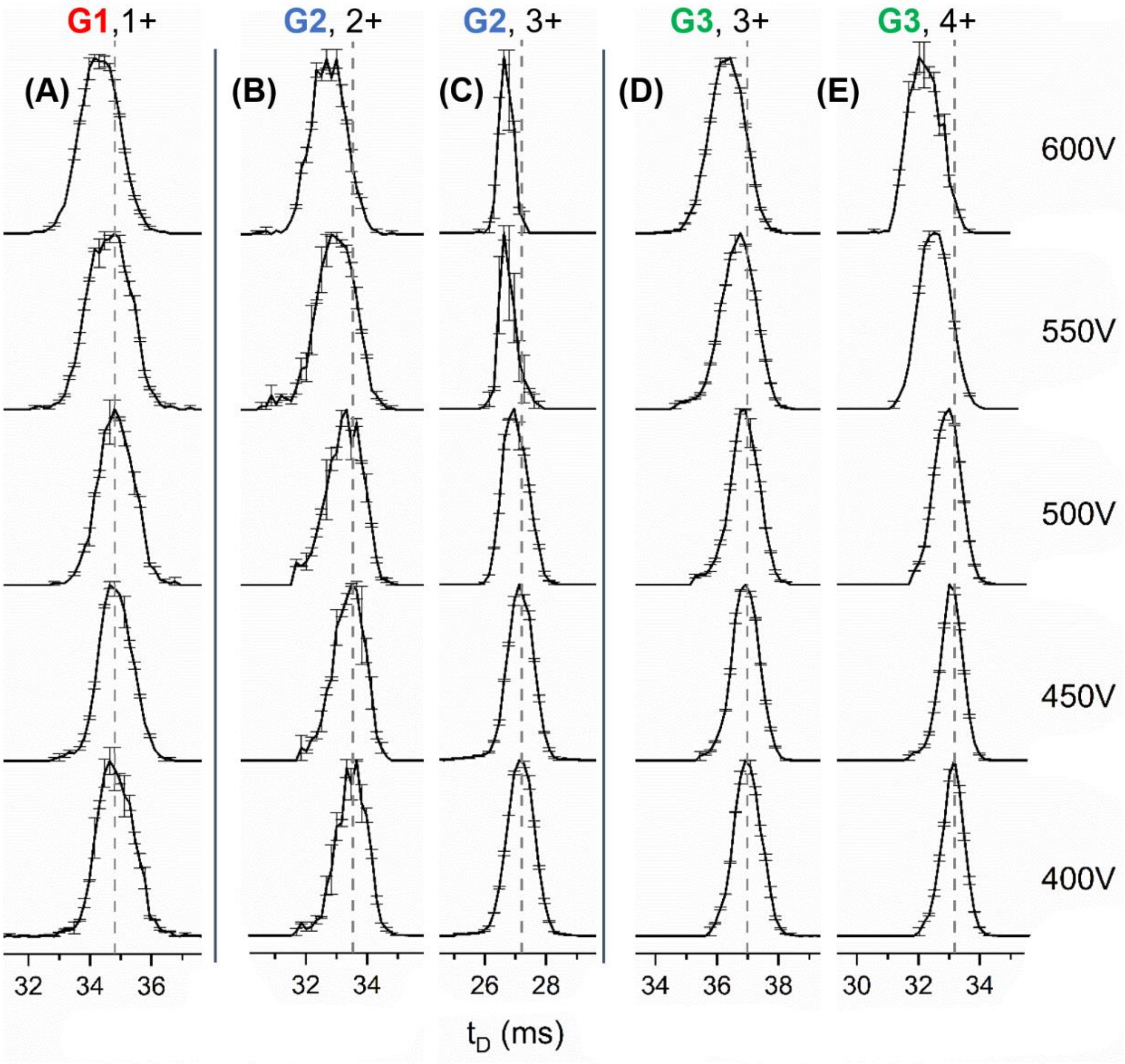

Figure S4. Arrival time distributions (ATDs) of the (A) charge state 1+ of G1, (B) charge state 2+ of G2, (C) charge state 3+ of G2, (D) charge state +3 of G3 and (E) charge state +4 of G3. ATDs were obtained for increasing activation voltages ranging from 400 to 600 V acquired on the Agilent 6560 IM-QToF in nitrogen. The vertical dashed lines represent the mean arrival time of the main feature at the lowest activation voltage (400 V) for each charge state. (2 µM in water for each dendrimer)

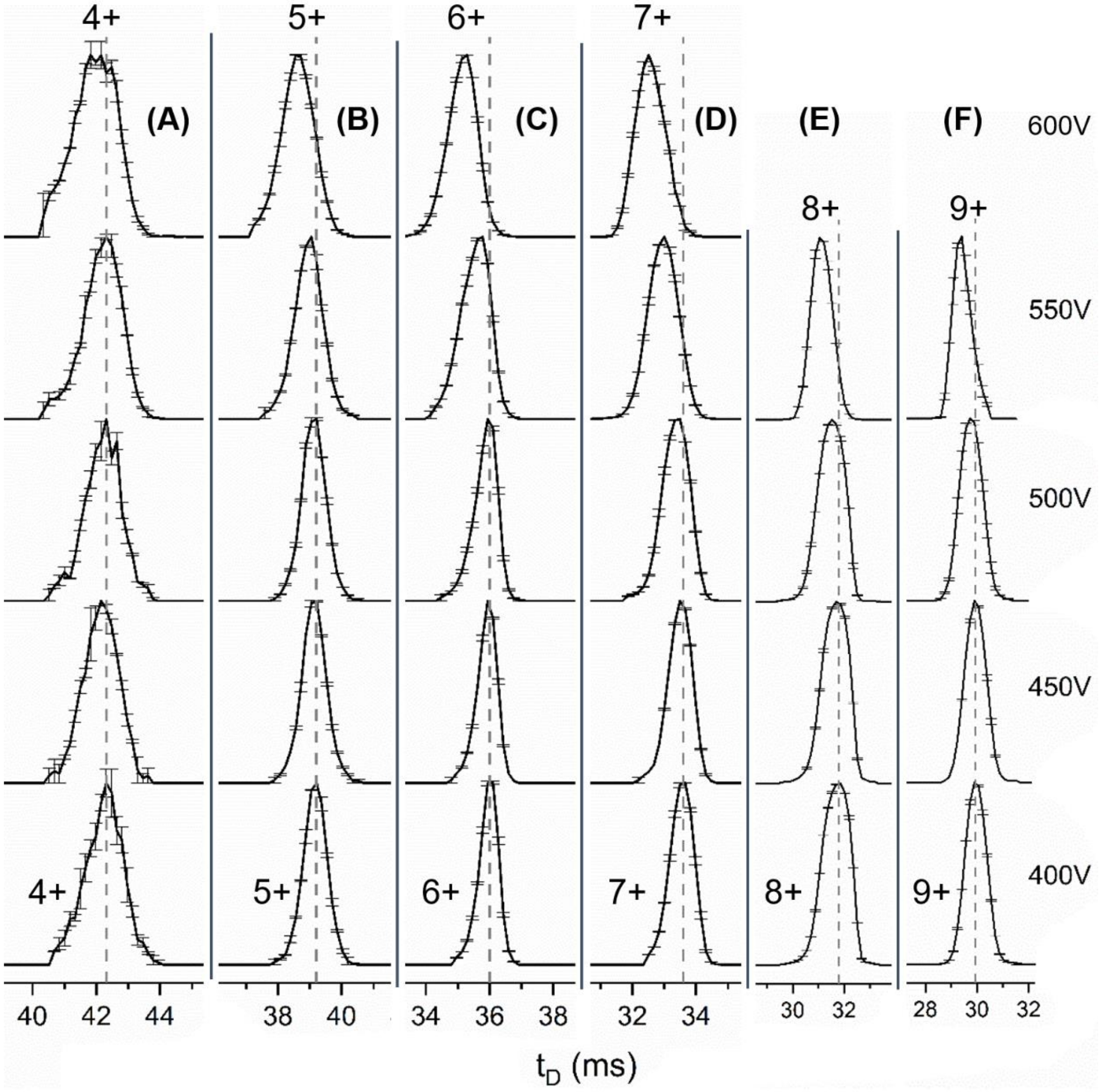

Figure S5. Arrival time distributions (ATDs) of dendrimer G4 between the charge states 4+ (A) and 9+ (F). ATDs were obtained for increasing activation voltages ranging from 400 to 600 V acquired on the Agilent 6560 IM-QToF in nitrogen. The vertical dashed lines represent the mean arrival time of the main feature at the lowest activation voltage (400 V) for each charge state. The increase in activation voltage causes a decrease in the mean arrival time for voltages beyond 500 V and more importantly for higher charge states. The 8+ and 9+ charge states were not detected at 600 V (2 µM in water for each dendrimer).

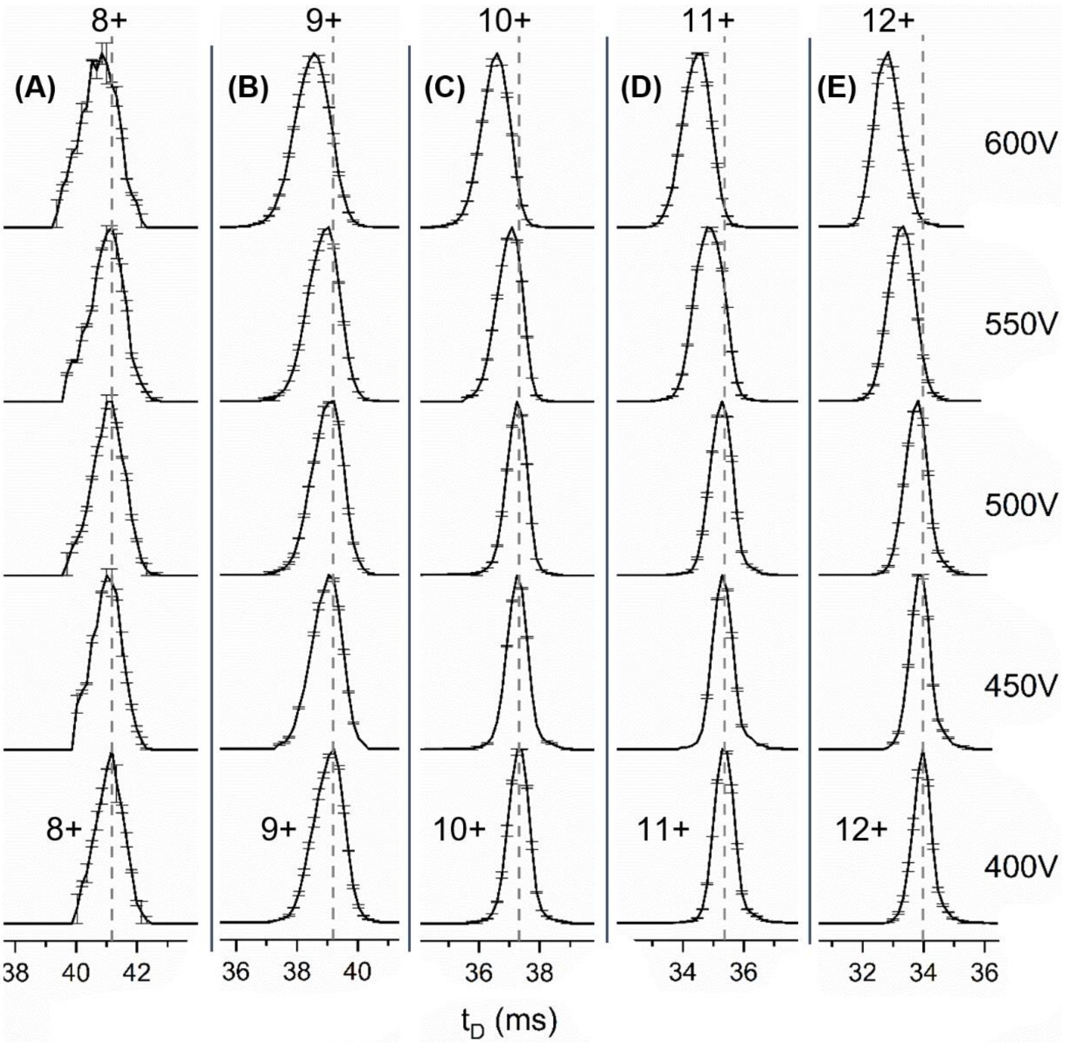

Figure S6. Arrival time distributions (ATDs) of dendrimer G5 between the charge states 8+ (A) and 12+ (F). ATDs were obtained for increasing activation voltages ranging from 400 to 600 V acquired on the Agilent 6560 IM-QToF in nitrogen. The vertical dashed lines represent the mean arrival time of the main feature at the lowest activation voltage (400 V) for each charge state. The increase in activation voltage causes a decrease in the mean arrival time for voltages beyond 550 V and more importantly for higher charge states. The lowest charge state shown (8+) retains the same mean average arrival time up until 550 V and slightly decreases at 600 V. A much larger decrease is observed at the same voltage for the highest charge state (12+), (2 µM in water for each dendrimer).

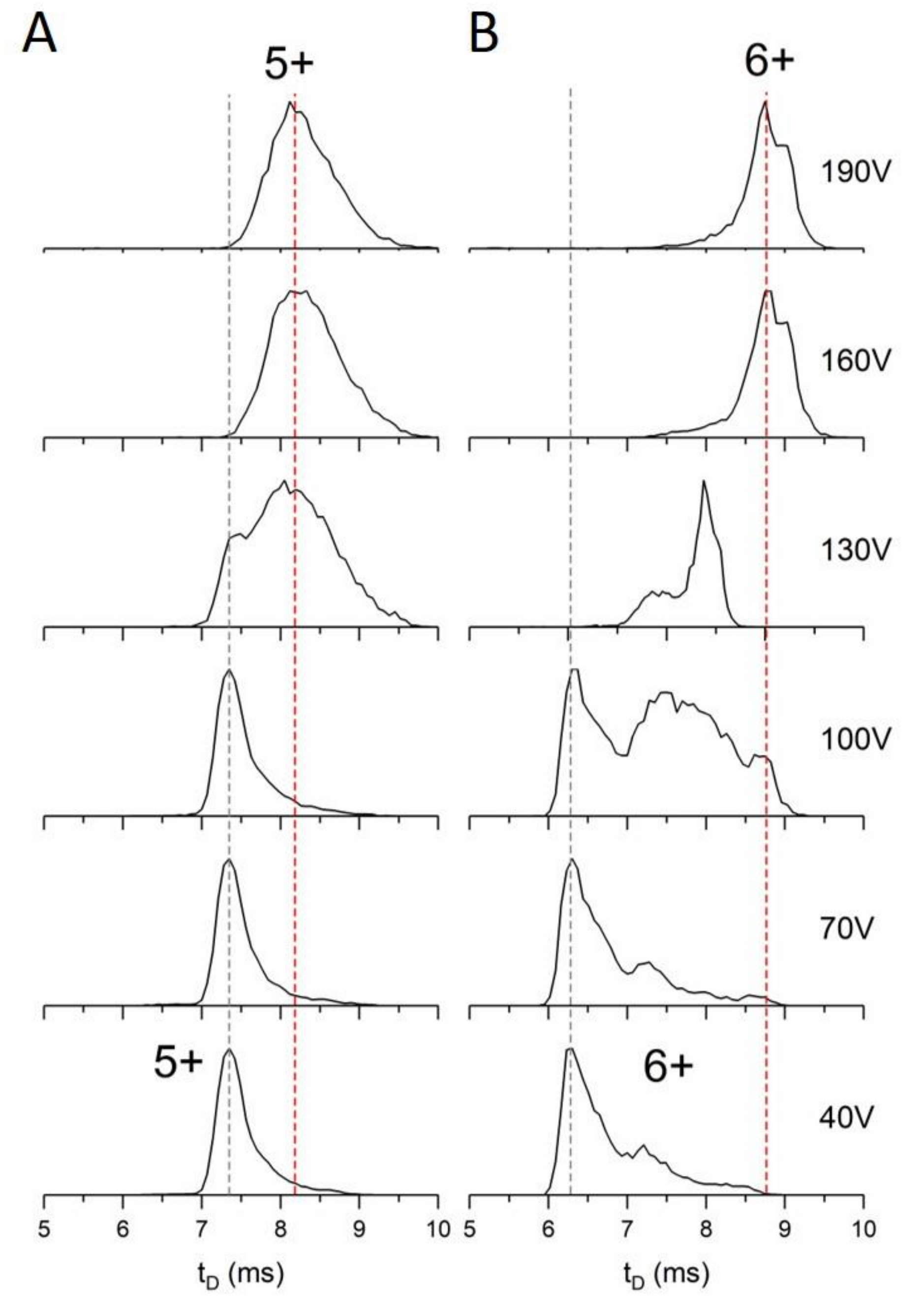

Figure S7. Arrival time distributions (ATDs) of the (A) 8+ and (B) 9+ native ubiquitin charge states obtained for increasing activation voltages ranging from 40 to 190 V acquired on the home-built VT-IM-MS instrument in helium (20µM in 50mM ammonium acetate). The grey vertical dashed lines represent the mean arrival time of the main distribution at the lowest voltage (20 V), while the red vertical dashed lines represent that of the main distribution at the highest voltage (190 V). Both charge states appear to unfold and/or fragment beyond 70 V activation as the arrival time distributions shift towards higher values.

Charge State	G5-Dendrimers $^{DT}CCS_{N2}$ (Å^2)	Ubiquitin $^{TW}CCS_{N2}$ (Å^2) Blend+Radial Function	Ubiquitin $^{TW}CCS_{N2}$ (Å^2) Power Law Function	Ubiquitin reference CCS_{N2} (Å^2)[5]	% Difference between Bush et al. data and BR/PL result
**7**	1512	1913	1919	1910	0.2%/0.5%
**8**	1655	1981	2029	1990	0.4%/2.0%
**9**	1774	2079	2120	2090	0.5%/1.5%
**10**	1878	2207	2246	2200	0.3%/2.0%
**11**	1955	2358	2398	2340	0.7%/2.5%
**12**	2041	2478	2515	2480	0.1%/1.4%
**13**	2131	2582	2616	2600	0.7%/0.6%
**14**	2227	-		-	-

Table S8. Ubiquitin $^{TW}CCS_{N2}$ calibrated by G5-Dendrimers using the power law[3] and the 'Blend+Radial' methods[4] each compared with reference CCS_{N2} data from Bush *et al.*[5]. Ubiquitin data was acquired in 5µM in water/methanol/acetic acid (49/49/2) on a Synapt G2-Si.

Charge State	$^{TW}CCS_{N2}$ (Å^2) (Synapt G2s)	Full Width at Half Maximum
**8**	1621.3 ± 0.9	49.9
**9**	1712.8 ± 0.3	59.3
**10**	1804.0 ± 0.4	46.0
**11**	1910.4 ± 0.6	31.1
**12**	2012.2 ± 0.2	29.7
**13**	2101.3 ± 0.1	41.6
**14**	2182.4 ± 0.2	47.5
**15**	2277.3 ± 0.3	58.2

Table S9. FWHM (Full width at half maximum) of Figure 2C, minimum for the 11+ and 12+ charge states indicate sub structure that is potentially more rigid/monodisperse for these two charge states.

	Fit function	R2 value
G1-N_2	-	-
G2-N_2	$^{DT}CCS_{N2}$ = 75.9z + 191.0	-
G3-N_2	$^{DT}CCS_{N2}$ = 106.8z + 340.3	-
G4-N_2	$^{DT}CCS_{N2}$ = 99.6z + 477.4	0.996
G5-N_2	$^{DT}CCS_{N2}$ = 99.0z + 868.4	0.995
G6-N_2	$^{DT}CCS_{N2}$ = 124.0z + 1106.0	0.983
G4-He	$^{DT}CCS_{N2}$ = 44.3z + 572.1	0.980
G5-He	$^{DT}CCS_{N2}$ = 78.2z + 656.4	0.993
G6-He	$^{DT}CCS_{N2}$ = 78.9z + 1175.9	0.991

Table S10. Fit function and R^2 value for $^{DT}CCS_{N2}$ and $^{DT}CCS_{He}$ from Figure 3.

References

(1) van Dongen, M. A.; Desai, A.; Orr, B. G.; Baker, J. R.; Holl, M. M. B. Quantitative Analysis of Generation and Branch Defects in G5 Poly(Amidoamine) Dendrimer. *Polymer* **2013**, *54* (16), 4126–4133. https://doi.org/10.1016/j.polymer.2013.05.062.

(2) Shek, P. Y. I.; Zhao, J.; Ke, Y.; Siu, K. W. M.; Hopkinson, A. C. Fragmentations of Protonated Arginine, Lysine and Their Methylated Derivatives: Concomitant Losses of Carbon Monoxide or Carbon Dioxide and an Amine. *J. Phys. Chem. A* **2006**, *110* (27), 8282–8296. https://doi.org/10.1021/jp055426k.

(3) Ruotolo, B. T.; Benesch, J. L. P.; Sandercock, A. M.; Hyung, S.-J.; Robinson, C. V. Ion Mobility–Mass Spectrometry Analysis of Large Protein Complexes. *Nat. Protoc.* **2008**, *3* (7), 1139–1152. https://doi.org/10.1038/nprot.2008.78.

(4) Richardson, K.; Langridge, D.; Dixit, S. M.; Ruotolo, B. T. An Improved Calibration Approach for Traveling Wave Ion Mobility Spectrometry: Robust, High-Precision Collision Cross Sections. *Anal. Chem.* **2021**, *93* (7), 3542–3550. https://doi.org/10.1021/acs.analchem.0c04948.

(5) Bush, M. F.; Hall, Z.; Giles, K.; Hoyes, J.; Robinson, C. V.; Ruotolo, B. T. Collision Cross Sections of Proteins and Their Complexes: A Calibration Framework and Database for Gas-Phase Structural Biology. *Anal. Chem.* **2010**, *82* (22), 9557–9565. https://doi.org/10.1021/ac1022953.
